# A prospective review of acute coronary syndromes in an urban hospital in sub-Saharan Africa

**DOI:** 10.5830/CVJA-2012-002

**Published:** 2012-07

**Authors:** Jay Shavadia, Gerald Yonga, Harun Otieno

**Affiliations:** Aga Khan University Hospital, Nairobi, Kenya; Aga Khan University Hospital, Nairobi, Kenya; Aga Khan University Hospital, Nairobi, Kenya

**Keywords:** acute coronary syndrome, clinical characteristics, sub-Saharan Africa

## Abstract

**Objectives:**

To determine the epidemiology of acute coronary syndromes (ACS) in sub-Saharan Africa.

**Methods:**

A prospective survey was carried out of all patients with a diagnosis of ACS who were admitted to the critical care unit of a tertiary teaching hospital over a 25-month period. Demographics, presentation, management and outcomes were subsequently recorded.

**Results:**

A total of 111 (5.1% of all hospitalisations) patients were recruited, with 56% presenting with ST-elevation myocardial infarction (STEMI) and the rest non-ST-elevation myocardial infarction (NSTEMI) or unstable angina (UA). Chest pain was the most common presenting symptom, and up to one-third of all STEMI patients did not receive any form of reperfusion therapy, primarily due to late presentation. As in the developed world, diabetes, hypertension and cigarette smoking still account for the most common predisposing risk-factor profile, and the mortality associated with ACS is about six to 10% in our unit.

**Conclusions:**

ACS, contrary to common belief, is increasingly more prevalent in sub-Saharan Africa, with similar risk profiles to that in the developed world. Late presentation to hospital is common and accounts for the increased mortality associated with this condition.

## Abstract

Sub-Saharan Africa has traditionally been viewed as the home of communicable diseases, and coronary artery disease was thought to be an extremely rare occurrence. Evidence for this emerged about 50 years ago when Florentin *et al*.^1^ reported no myocardial infarctions in 182 consecutively performed autopsies among Ugandans. At about the same time, Shaper^2^ reported no coronary artery disease in 100 Samburu elders, based on a physical examination and electrocardiography. These were about the only tools available to make an ante-mortem diagnosis of ischaemic heart disease at the time.

Smaller studies from other parts of East and central Africa consistently reported a paucity of ischaemic heart disease with little evidence of the traditional risk factors of hypertension, hyperlipidaemia and obesity.^3^-^6^ No information regarding diabetes mellitus and smoking status has been reported in these groups.

The last few decades have witnessed considerable transition in epidemiology and with it came a change in the pattern of disease. Increasing urbanisation and changing lifestyle profiles have triggered an exponential rise in the frequency of the historically absent traditional coronary artery disease risk factors in black Africans.^7^-^11^

In addition to the unfinished agenda on communicable diseases, health planners in African countries are now being faced with a rising burden of non-communicable diseases.^12^ Cardiovascular disease, particularly coronary artery and cerebrovascular disease are on the rise, and will soon represent the bulk of the morbidity and mortality in this non-communicable medical disease bracket. We set out to define the demographics, presentation and outcomes of patients admitted with an acute coronary syndrome (ACS) at the Aga Khan University Hospital, Nairobi (AKUHN), a tertiary-care hospital running a cardiac catheterisation laboratory with a cardiac intervention programme in Nairobi, Kenya.

## Methods

A prospective survey was done of all consecutive patients with a diagnosis of ACS admitted to AKUHN between April 2008 and May 2010. All patients had their data regarding the study variables entered into the study questionnaire after giving informed consent. ACS was defined as patients admitted with ST-elevation myocardial infarction (STEMI), non-ST-elevation myocardial infarction (NSTEMI) and unstable angina (UA).

STEMI was diagnosed if ST-segment elevation at the J point of ≥ 1 mm occurred in any location or a new left bundle branch block (LBBB) was found on the electrocardiogram (ECG) with biochemical evidence of myocyte necrosis.^13^ NSTEMI was diagnosed in patients with biochemical indication of myocyte necrosis without new ST-segment elevation or LBBB on ECG.^13^

Markers of myocyte necrosis utilised in our study included an elevated (exceeding the 99th percentile of a reference control group) serum troponin I and CK-MB level.^13^ Unstable angina was considered to be present in patients with ischaemic symptoms and no elevation in CK-MB or troponin levels, with or without ECG changes suggestive of ischaemia (e.g. ST-segment depression or new T-wave inversion).^14^

All patients with a confirmed diagnosis of ACS were prospectively recruited from either the intensive care or the high dependency units, where critically ill patients requiring continuous monitoring were admitted. Data were entered into a questionnaire within 24 hours of the patient’s hospitalisation. The questionnaire was subdivided into the following components:

• Demographics: age (years), gender, height (cm) and weight (kg) were recorded and a body mass index (kg/m^2^) was subsequently computed.

• Clinical presentation: patients presenting with symptoms (chest pain, syncope, dyspnoea, cardiac arrest, other), systolic blood pressure (mmHg), heart rate (beats/minute), Killip class (I, II, III or IV) and time from symptom onset to hospital presentation (minutes).

• ECG and laboratory findings: predominant rhythm, ST-segment and T-wave changes were recorded according to the ECG site of myocardial involvement. Serum chemistries included biomarkers of myocyte necrosis (CK-MB, troponin I levels), fasting lipid profile including total cholesterol, high-density lipoprotein, low-density lipoprotein, blood glucose (mmol/l) levels and calculated creatinine clearance (ml/min) at presentation using the Cockcroft-Gault formula.

• Risk factor assessment: a record of pre-existing type 2 diabetes mellitus, hypertension, cigarette smoking status, history of dyslipidaemia, family history of premature coronary artery disease, prior anginal episodes, myocardial infarction, percutaneous coronary interventions (PCI) or coronary artery bypass graft (CABG) operations and prior history of stroke/cerebrovascular accident was obtained from the patients’ history and medical notes.

• Diagnosis and management: all patients were sub-grouped into two classes: STEMI and NSTEMI/UA, according to the ECG and serum biomarkers of myocyte necrosis. Management strategies were analysed in terms of reperfusion therapy and adjunctive therapies administered during hospitalisation.

- acute reperfusion strategy and modality utilised (none, fibrinolysis, PCI) for the STEMI subgroup.

- adjunctive therapy including the use of anti-thrombotic therapy, antiplatelet agents, beta-blockade, angiotensin receptor blockade and lipid-lowering agents.

All patients who underwent diagnostic coronary angiography alone or in addition to PCI had their angiographic findings and procedural details recorded.

• Outcomes: in-hospital outcomes were recorded according to the proportion of patients alive and complications (heart failure, major bleeding, post-myocardial infarction (MI) re-infarction and sudden death) at discharge.

## Statistical analysis

All patients were grouped into either STEMI or NSTEMI/UA and data were subsequently analysed using SPSS version 15.0. All continuous variables are expressed as mean ± standard deviation. Categorical variables are expressed as percentages. Statistical comparisons between subgroups were performed using the Chi-square test, the Fisher exact test for categorical variables, and the Student’s *t*-test for continuous variables, and regression analyses as appropriate, using SPSS.

## Results

A total of 2 156 mainly medical patients were admitted to the high dependency and intensive care units between April 2008 and May 2010, 111 (5.1%) of whom had a confirmed diagnosis of ACS. Fifty-six per cent (62) of the patients had a diagnosis of STEMI with the rest being NSTEMI/UA (44%). The baseline characteristics and risk-factor profiles of these groups are shown in Tables 1 and 2.

**Table 1.** Baseline Characteristics Of Patients Presenting With ACS

	*STEMI (n = 62)*	*NSTEMI/UA (n = 49)*	p
Mean age (years)	63.3 ± 13.0	64.5 ± 15.2	NS
Male gender (%)	80.6	69.4	NS
BMI (kg/m^2^)	26.7 ± 3.9	27.3 ± 4.8	NS
Chest pain as presenting symptom (%)	83.9	69.4	NS
Mean time to presentation (hours after symptom onset)	12.9 ± 17.3	29.3 ± 52.8	0.02
Mean systolic BP at presentation (mmHg)	133 ± 35	139 ± 29	NS
Mean heart rate at admission (bpm)	85 ± 22	82 ± 18	NS
Killip class (%)			
I	88.7	89.8
II	11.3	4.1
III	0	6.1
IV	0	0
ECG rhythm at presentation (%)			
Sinus	91.9	93.9
Atrial fibrillation	3.2	4.1
Other	4.8	2.0
Random glucose at admission (mmol/l)	8.9 ± 5.0	6.9 ± 3.2	0.009
Creatinine clearance (ml/min)	68.3 ± 31.3	64.2 ± 31.3	NS

bpm = beats per minute, NS = non-significant.

**Table 2.** Cardiovascular Risk Factor Profile

	*STEMI (n = 62)*	*NSTEMI/UA (n = 49)*	p
History of diabetes mellitus (%)	38.7	34.7	NS
History of hypertension (%)	46.8	51.0	NS
Current smoker (%)	24.2	22.4	NS
Family history of CAD (%)	16.1	16.3	NS
Dyslipidaemia (%)	9.7	20.4	NS
Prior angina (%)	6.5	14.3	NS
History of CABG (%)	1.6	0	NS
Previous PCI (%)	3.2	2.0	NS
Previous MI (%)	6.5	12.2	NS
History of stroke (%)	0	4.1	NS
Total cholesterol (mmol/l)	5.4 ± 1.4	5.5 ± 1.6	NS
LDL-C (mmol/l)	3.3 ± 1.0	3.0 ± 1.2	NS
HDL-C (mmol/l)	1.1 ± 0.5	0.9 ± 0.2	0.018

CAD = coronary artery disease, PCI = percutaneous coronary interventions, CABG = coronary artery bypass graft, MI = myocardial infarct, LDL-C = low-density lipoprotein cholesterol, HDL-C = high-density lipoprotein cholesterol.

**Fig. 1.**
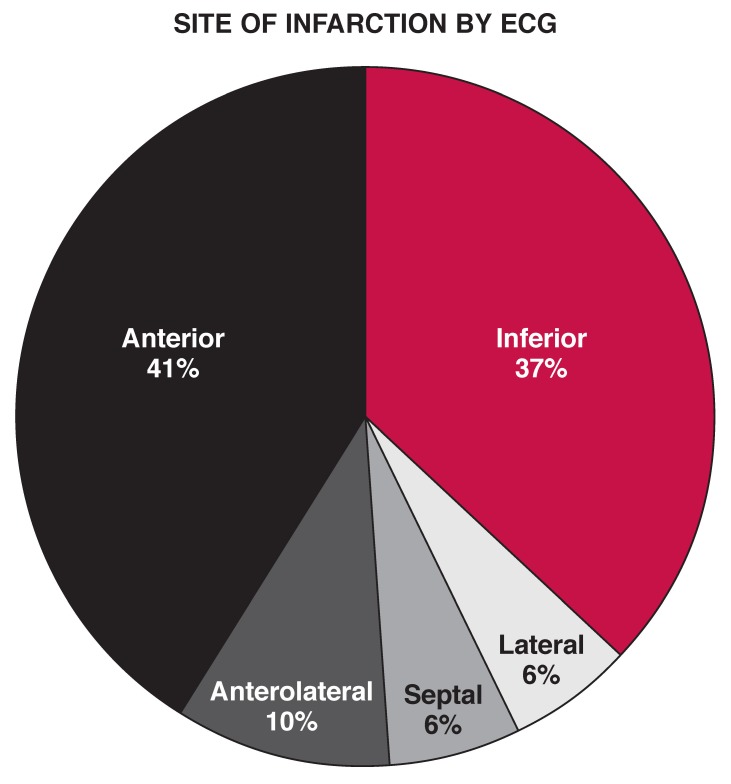
Site of myocardial involvement in patients with STEMI.

About 58% (36) of the STEMI patients and 72% (36) of the NSTEMI/UA patients had one or two of the risk factors described above, while 14% (nine) and 16% (eight) of the STEMI and NSTEMI/UA arms, respectively, had none of the above traditional risk factors at presentation. Over 85% (52) of the patients in the STEMI subgroup had a TIMI risk score between two and eight, while 69.4% (34) in the NSTEMI/UA subgroup had a score of two and three at admission. The site of myocardial involvement in patients with STEMI is shown in Fig. 1.

The current ACS management strategy for STEMI at AKUHN is prompt fibrinolysis within 30 minutes, and primary PCI whenever deemed logistically feasible within 90 minutes of arrival. Fifty-five per cent (34) of patients presenting with STEMI received fibrinolysis, with the majority receiving tenecteplase. Thirteen per cent (eight) of the patients underwent primary PCI because they had an absolute contraindication to fibrinolysis. Over 30% (20) of the patients did not receive any form of reperfusion, primarily due to late presentation at hospital.

The mean door-to-needle time in our study was 47 ± 48 minutes while the mean door-to-balloon time was 84 ± 67 minutes. Patients with STEMI had an average hospital stay of 5.2 ± 3.9 days while patients with NSTEMI were in hospital for an average of 4.7 ± 2.8 days (*p* = 0.54).

Just under half (48%) of the STEMI subgroup, and 49% of the NSTEMI/UA subgroup underwent coronary angiography, with the following findings: in the STEMI group, the left anterior descending artery was the culprit vessel in 40% of the patients, and 33% had the right coronary and 7% the left circumflex arteries as the culprit arteries, respectively. Twenty per cent (six) of the patients who had coronary angiography in the STEMI subgroup had multi-vessel disease. These patients were maintained on optimal medical therapy, and none underwent multi-vessel PCI at the time of their STEMI presentation.

As anticipated, over half (56%) the patients in the NSTEMI/UA subgroup had angiographically double- or triple-vessel disease. Of this subgroup, 29% (14) were deemed appropriate, and referred for surgical revascularisation, since this service was not being offered at our institution. Data on the surgical outcomes of these patients were not available, as a majority were referred to overseas centres for surgery. An additional 12% (six) underwent staged PCI. Data on the revascularisation strategy for the remainder of the NSTEMI/UA subgroup were unknown.

Ninety per cent (56) of the STEMI patients were alive at discharge, compared to 94% (46) of the NSTEMI/UA patients. Of all the patients in the study, 10% (11) developed clinical heart failure, 1.8% (two) major bleeding (according to TIMI 7, 8 11B criteria)^15^ and 1.8% (two) had other complications. One of the patients in the major bleed subgroup had intracranial haemorrhage and was discharged with severe disability (modified Rankin scale 5). None of the study patients developed post MI re-infarction.

## Discussion

Contrary to earlier statistics that ACS is rare in sub-Saharan Africa, our study illustrates that nearly 5% of all the high dependency and intensive care admissions in our hospital are due to an acute coronary event. This clearly illustrates the changing prevalence of ischaemic heart disease in eastern Africa in comparison with earlier data.

Over half of the patients admitted in our study had a diagnosis of STEMI. This contrasts to larger databases, such as the GRACE registry from North America and Europe, which reported 30% of their total number to be STEMI.^16^ This difference could primarily be due to the small numbers involved in our study.

The mean age of 63–64 years at presentation in our two study subgroups was comparable, however the mean age in our study was about a decade older compared to that in the INTERHEART Africa cases.^11^ There was an overwhelming male predominance in both subgroups in our study, similar to data from the INTERHEART Africa study.^11^ This could be due to the already established risk the male gender confers, or represents the health-seeking behaviour of the male gender in our country.

Diabetes mellitus, hypertension and current smoking, akin to data from other parts of the world, comprised the commoner risk factors for the development of CAD in our study. Data from INTERHEART Africa showed that the traditional cardiovascular risk factors (current/former tobacco smoking, diabetes, hypertension, obesity and dyslipidaemia) relating to ischaemic heart disease in the West also account for nearly 90% of the risk for an initial MI in Africans.^11^ Our data reaffirm the fact that the risk-factor profile for the development of an MI may be no different in a black African, and that the rise in ischaemic heart disease is probably due to the increasing prevalence of these traditional risk factors in sub-Saharan Africa.

Chest pain was the commonest presenting symptom. However, one in every five (20%) patients in our study presented with a symptom other than chest pain. These atypical presentations included epigastric pain, dyspnoea or syncope. It is this cohort of patients who are likely to be improperly triaged in the emergency room, only to present later as a medical catastrophe. Emphasis should therefore be laid on these atypical presentations in patients at risk for ACS presenting to the triage facilities of the emergency departments in this part of the continent.

It was surprising to note that patients with STEMI took over 13 hours, while NSTEMI more than twice as long to present to the emergency room from the onset of their symptoms. Since these patients had had on-going symptoms for over 12 hours, the reasons for late presentation will be important to evaluate in the future. This information may help improve patient education in the community on the symptoms of ACS, and hopefully improve outcomes in such patients in our setting by earlier administration of reperfusion therapy in the hospital.

Reperfusion strategies in STEMI have clearly been outlined in many international management guidelines.^17^ However, the unavailability of PCI facilities and trained personnel, as well as cost implications are the major causes of deviance from these guidelines in our set up. This is highlighted by the low rates of primary PCI and prolonged door-to-balloon times in our study, with the majority of the eligible patients receiving fibrinolysis. An even more grim observation is the failure of one-third of all STEMI patients to receive any form of reperfusion due to late presentation to hospital.

The presumed reasons for this could be several, but include challenges at multiple tiers in the healthcare delivery system, including patient awareness of symptoms of myocardial ischaemia, the risks of delayed medical care for STEMI, lack of pre-hospital transportation systems and ECGs, and failure to diagnose STEMI on initial ECG by poorly exposed healthcare personnel. All these areas deserve future evaluation and study in order to reduce the time of presentation to hospital for ACS.

In our study, the majority of patients received loading doses of aspirin, clopidogrel and enoxaparin as the anticoagulant of choice. The anticoagulant doses were calculated on approximate body weight; however, no record of dose adjustment on the estimated creatinine clearance rate was noted. Over half the patients in both arms of the study received ACE inhibitors or ARBs and over three-quarters received a beta-blocker. The adherence rates to guideline-based therapies for ACS were similar to centres in North America and Europe, reflecting similarities in practice for adjunctive therapies for ACS.^18^,^19^

GP II_b_/III_a_ inhibitors were more commonly utilised in the NSTEMI subgroup and this group had more coronary angiographies compared to the STEMI subgroup. As anticipated, patients with NSTEMI had more double- and triple-vessel disease. However, the lower rates of total revascularisation in this subgroup were due to associated patient comorbidities tilting the risk–benefit balance, cost of surgical revascularisation, and the lack of an effective surgical revascularisation team, hence treating physicians opting for a staged PCI procedure or medical treatment alone in these patients with multi-vessel coronary artery disease.

The average length of hospital stay in the study was five days, accounting to direct hospital costs of approximately Kenya Shillings 400 000 (US$ 6 000; 1 US$ = KSh 70). This includes the cost of admission to the critical care unit and ward stay, laboratory and radiological investigations, cardiac catheterisation and stenting, and drug therapy in hospital. Strategies to lower costs and make this superior reperfusion strategy more available will need to be addressed.

The in-hospital mortality rates were 9.7 and 6.0% in the STEMI and NSTEMI subgroups, respectively. These figures are much higher than those reported in the GRACE and NRMI registries. This reflects the delayed presentation time of our STEMI patients. One-year data for the UA/NSTEMI patients will need to be evaluated to see the long-term mortality trends.

The beginning of every good policy is generation of good local data. This article serves as an important first step in understanding the characteristics of patients with ACS in sub-Saharan Africa. AKUHN receives patients from all over the East African region and reflects practice in a modern sub-Saharan tertiary referral hospital. Improved public and patient education on the early recognition of myocardial ischaemia, development of an integrated pre-hospital emergency medical transportation system and reducing costs of reperfusion therapy may facilitate reduction of mortality associated with ACS.

This observational study was limited by its small numbers and therefore was unable to be conclusive. We were also unable to make strong comparisons between the differences in presentation, management and outcomes of patients with STEMI compared to NSTEMI.
